# Recent advances in understanding the human host immune response in tuberculous meningitis

**DOI:** 10.3389/fimmu.2023.1326651

**Published:** 2024-01-09

**Authors:** James R. Barnacle, Angharad G. Davis, Robert J. Wilkinson

**Affiliations:** ^1^ The Francis Crick Institute, London, United Kingdom; ^2^ Department of Infectious Disease, Imperial College, London, United Kingdom; ^3^ Centre for Infectious Diseases Research in Africa, Institute of Infectious Disease and Molecular Medicine, University of Cape Town, Observatory, South Africa

**Keywords:** tuberculous meningitis (TBM), tuberculosis, *Mycobacterium tuberculosis*, extrapulmonary tuberculosis (EPTB), immune response, host-directed therapy, immunity

## Abstract

Tuberculous meningitis (TBM), the most severe form of tuberculosis, causes death in approximately 25% cases despite antibiotic therapy, and half of survivors are left with neurological disability. Mortality and morbidity are contributed to by a dysregulated immune response, and adjunctive host-directed therapies are required to modulate this response and improve outcomes. Developing such therapies relies on improved understanding of the host immune response to TBM. The historical challenges in TBM research of limited *in vivo* and *in vitro* models have been partially overcome by recent developments in proteomics, transcriptomics, and metabolomics, and the use of these technologies in nested substudies of large clinical trials. We review the current understanding of the human immune response in TBM. We begin with *M. tuberculosis* entry into the central nervous system (CNS), microglial infection and blood-brain and other CNS barrier dysfunction. We then outline the innate response, including the early cytokine response, role of canonical and non-canonical inflammasomes, eicosanoids and specialised pro-resolving mediators. Next, we review the adaptive response including T cells, microRNAs and B cells, followed by the role of the glutamate-GABA neurotransmitter cycle and the tryptophan pathway. We discuss host genetic immune factors, differences between adults and children, paradoxical reaction, and the impact of HIV-1 co-infection including immune reconstitution inflammatory syndrome. Promising immunomodulatory therapies, research gaps, ongoing challenges and future paths are discussed.

## Introduction

Tuberculous meningitis (TBM), the most severe form of tuberculosis, carries a mortality of over a quarter despite antibiotic therapy, and over half of survivors are left with neurological disability (1). In HIV-1 coinfection, mortality rates can approach 50% (2). In tuberculin-sensitised animals, death has been shown to occur after meningeal inoculation of dead bacilli (3) and it is recognised that a dysregulated and excessive host immune response contributes significantly to poor outcome. Host-directed therapies (HDT) are a promising tool to modulate this immune response and improve outcomes. Over the past 20 years the only evidence based adjunctive HDT in TBM has been corticosteroids (4). However, corticosteroids remain a blunt and poorly understood immunomodulator in TBM. Recently, studies have shown that corticosteroids may not reduce mortality or neurologic outcomes in people living with HIV (PLWH), and amongst HIV-uninfected patients, not all patients may benefit depending on their genotype (5, 6).

There is therefore an acute need for novel HDT and a comprehensive understanding of the host immune response to TBM is essential in developing new therapies or re-purposing existing ones. Challenges with accessing the site of disease and a lack of *in vivo* and *in vitro* models have been partially offset by recent developments in multiomics technology, allowing large amounts of data to be generated from limited clinical samples. This review examines recent advances in our understanding of human host immune responses in TBM beginning with *Mycobacterium tuberculosis* (*M. tuberculosis*) entry into the CNS, then moving to CNS barrier dysfunction, the innate and adaptive responses, and metabolomic and genetic aspects. It draws from the wider literature on tuberculosis and neurological infection to identify gaps and opportunities in TBM research. Promising HDT, future challenges and a path forward are discussed.

## The host immune response against TBM

### The CNS immune system in health

**Figure 1:**
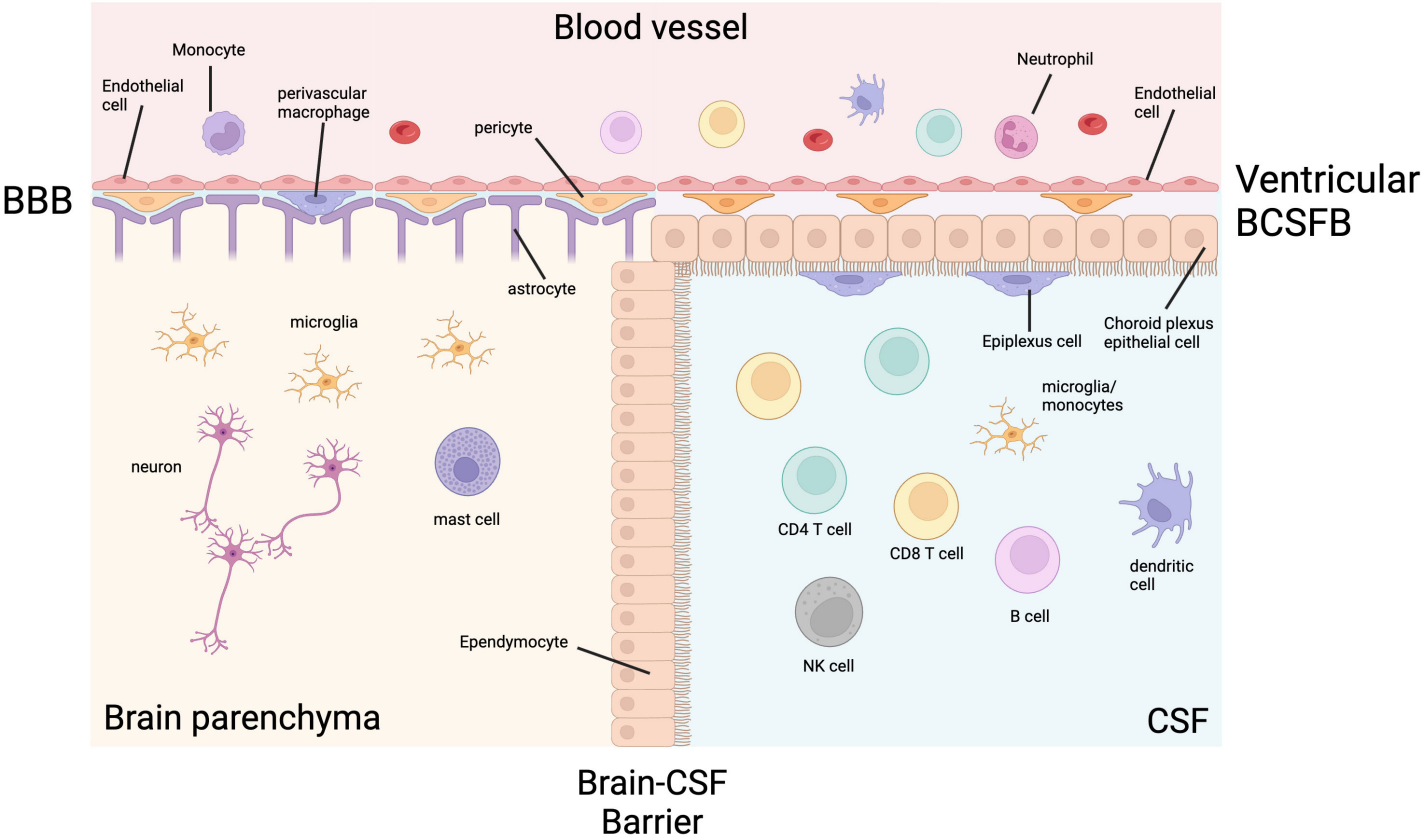
The ventricular CNS barriers in health. Cerebrospinal fluid (CSF) and the brain parenchyma are separated from the systemic blood circulation by the blood-brain barrier (BBB) and blood-CSF barrier (BCSFB). The BBB is made up of a luminal endothelial cell layer, a basement membrane, a perivascular space containing pericytes and perivascular macrophages, and layer of astrocytic foot processes. The ventricular BCSFB is made up of an endothelial layer, a stromal layer containing pericytes, choroid plexus epithelial cells, and epiplexus cells (macrophages). The brain parenchymal immune cells are limited to microglia, macrophages and mast cells. The CSF is less privileged and contains lymphocytes, as well as lower numbers of other immune cells. Created with BioRender.com.

To understand the immunopathogenesis of TBM, physiological CNS immunity must be understood. The only immune cells found in the brain parenchyma in health are microglia, mast cells and macrophages (7). This immune privilege is maintained by the blood-brain barrier (BBB) and the brain-cerebrospinal fluid (CSF) barrier (BCSF) (**Figure 1**). The BBB is a collection of structural, transport and metabolic barriers maintained by endothelial cells bound together with tight junctions and adherens junctions lining the blood vessels, which are enveloped by pericytes and astrocytes (8). These endothelial cells have low rates of transcytosis, low numbers of leukocyte adhesion molecules and specific efflux transporters (9).

CSF is less immune privileged than the brain parenchyma and has a more diverse immune cell composition physiologically, but it is still protected from the systemic circulation. In the ventricles, the BCSFB is formed by tight junctions between epithelial cells of the choroid plexus. Epiplexus macrophages, also known as Kolmer cells, adhere to the CSF side of the epithelial layer and have phagocytic and antigen-presenting properties. In the meningeal and cisternal subarachnoid space, blood vessels have a similar structure to the BBB, except they lack the surrounding pericytes and astrocytes. The subarachnoid space is separated from the dura by arachnoid barrier cells containing tight junctions forming a barrier between the fenestrated vasculature of the dura and the CNS (10, 11). Despite these robust defences, *M. tuberculosis* can enter the CNS and cause TBM, spinal arachnoiditis and tuberculomas.

### Entry into the CNS

TBM follows systemic infection with *M. tuberculosis*. After inhalation of aerosolised *M. tuberculosis* and infection of alveolar macrophages, a cytokine response induces migration of dendritic cells to local lymph nodes. Dendritic cells (DC) present antigen and stimulate T-helper 1 (Th1) cell differentiation. Alongside cytotoxic and regulatory functions, these differentiated Th1 cells release TNF, IFNγ and other cytokines at the site of infection which activate macrophages to contain *M. tuberculosis* and form granulomas.

**Figure 2:**
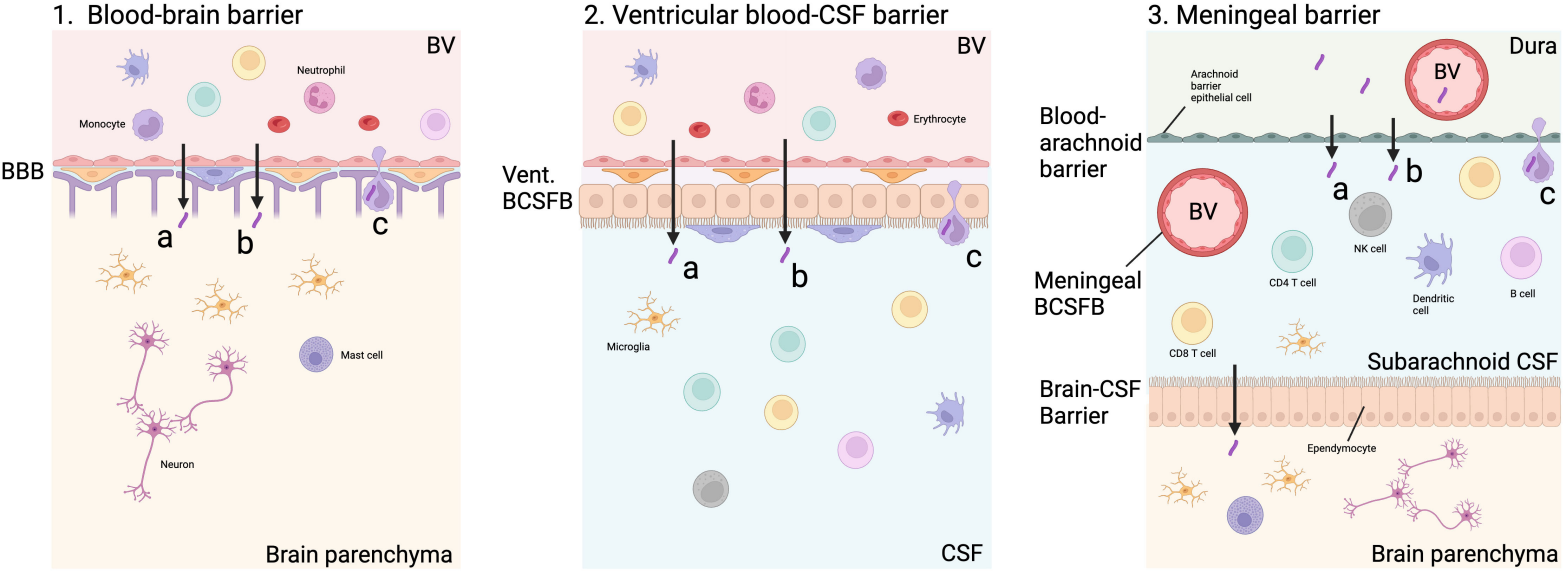
Possible routes of *M. tuberculosis* entry into CNS. *M. tuberculosis* may enter the brain parenchyma directly via capillary blood flow through the blood-brain barrier (BBB, Figure 2.1). This may be via paracytosis **(A)**, transcytosis **(B)** or within an infected cell **(C)**. *M. tuberculosis* may also pass through the blood-CSF barrier lining the ventricles, which contains an epithelial choroid plexus layer along with an endothelial layer through the same mechanisms (Figure 2.2). In the meninges, the blood-arachnoid barrier separates the unfiltered interstitial fluid and blood from the dura from the sub-arachnoid space within the CNS via an epithelial layer of arachnoid barrier cells. The arachnoid barrier cells are poorly vascular, and exchange is likely to be minimal. In addition, blood vessels (BV) passing through the subarachnoid space represent another blood-CSF barrier which does not contain a choroid plexus layer. *M. tuberculosis* in the subarachnoid space may be able to pass across the brain-CSF barrier formed by an ependymal layer into the brain parenchyma (Figure 2.3). Created with BioRender.com.

In those with an inadequate primary immune response, or later immune compromise, control of *M. tuberculosis* in the lungs is lost and pulmonary or systemic spread may occur. The exact mechanism of *M. tuberculosis* infiltration into the CNS remains uncertain. It is thought that bacilli cross the BBB by transcytosis, paracytosis or within phagocytic immune cells (‘Trojan Horse’) following haematogenous spread (**Figure 2**). An *in vitro* study showed that *M. tuberculosis* was able to cross a model BBB significantly more efficiently than non-pathogenic *M. smegmatis* via actin rearrangement of EC (12). A murine model found that CNS invasion was attenuated in five *M. tuberculosis* mutants, including *Rv0986*, which forms a transporter thought to be involved in cell adhesion and entry, and *Rv0931c* which encodes a serine-threonine protein kinase thought to help *M. tuberculosis* adapt to unfavourable environments, such as the CNS (13). In a zebrafish model, *M. marinum* was able to cross the BBB within infected phagocytes, as well as via ESX-1-dependent invasion of EC (14). These studies suggest that *M. tuberculosis* may use a combination of methods and virulence factors to pass through the BBB and survive in the CNS environment.

Following infiltration into the CNS, a leptomeningeal or cortical focus known as a ‘Rich focus’ is established. Rich and McCordock were sceptical of the link between the ‘Rich focus’ and miliary TB citing examples of TBM without miliary spread and cortical lesions that preceded miliary spread in post-mortems (15), however, it is now widely believed that the primary route of CNS infiltration is haematogenous, although bacillaemia may not always be recognised (16). The granulomatous lesion is likely to progress through a non-necrotising phase characterised by activated epithelioid macrophages, a necrotising gummatous phase with central necrosis and reticulin fibres, and finally a necrotising abscess with leukocytoclastic neutrophils which ruptures into the subarachnoid space (17). Unlike the brain parenchyma, subarachnoid blood vessels are not supported by astrocytic foot processes and are therefore more permeable to bacilli and immune cells, potentially explaining why TBM is most often localised to the leptomeninges and subpial cortex; deep parenchymal granulomas are rarely seen (17).

There is a lack of research and understanding about other CNS barriers that may be entry points for *M. tuberculosis* and immune cells. The initial point of *M. tuberculosis* entry may be across the ventricular BCSFB, meningeal BCSFB or arachnoid barrier, rather than the BBB (**Figure 2**) (18). Classically, the BCSFB is described as having a choroid plexus epithelial cell layer which is found predominantly, if not exclusively, lining the ventricles. However, in the meninges, blood vessels pass through the sub-arachnoid CSF creating another BCSFB (19). Another meningeal barrier is that of the epithelial arachnoid barrier cells. Compared to other layers, this layer is thought to play a minimal role in exchange because of its avascular nature and lower surface area (18). After the rupture of the initial granuloma, the leukocyte infiltration into the CSF seen in TBM is unlikely to come via the brain parenchyma following BBB infiltration, but rather across the BCSFB. These meningeal and cisternal barriers are poorly understood and have not been modelled in TBM. The factors influencing the permeability of these barriers may play an important part in immunopathology and should be investigated.

### Microglial activation releases cytokines and initiates inflammatory response

Microglia are the principal cells infected by *M. tuberculosis* (20). Direct infection of neurons and astrocytes may occur to a lesser extent (21). Given the established roles of both these latter cell types in the immune axis, this is likely to influence the host immune response in TBM. Microglia recognise *M. tuberculosis* via pattern recognition receptors (PRR) such as toll-like receptors (TLR). Internalisation of *M. tuberculosis* may be dependent on CD14, a monocyte differentiation antigen, demonstrated by a study which showed anti-CD14 monoclonal antibodies and soluble CD14 ligand reduced the uptake of non-opsonised bacilli by foetal microglia by 64% and 62% respectively (22), but this was not reproduced in human monocytes, monocyte-derived macrophages or alveolar macrophages. CD14 expression on these cells however increases in *M. tuberculosis* infection (23).

Activated microglia secrete cytokines which mediate immune cell recruitment and play an essential role in host defence but may also contribute to excess inflammation. Microglia infected with *M. tuberculosis* have been shown to secrete TNF, C-C motif chemokine ligand 2 (CCL2), CCL5, C-X-C motif chemokine ligand 10 (CXCL10), IL-1α, IL-10, IL-12p40, granulocyte colony-stimulating factor (G-CSF) and granulocyte-macrophage colony-stimulating factor (GM-CSF) *in vitro*, demonstrating the role of microglia in escalating the initial immune response through stimulation, activation and chemoattraction of other leukocytes (21, 24). The IFNγ-inducible chemokines CXCL9 and CXCL10 are secreted by microglia and macrophages, as well as astrocytes in the case of CXCL10, and are important in T cell and NK cell recruitment. CSF CXCL9 and CXCL10 levels are significantly increased in TBM compared to other neurological infections and decrease with treatment (25). The chemokines CCL2 and CCL5 recruit leukocytes to the CNS which may contribute to immunopathology, but CCL2 has also been shown to reduce neuronal death following N-methyl-D-aspartate (NMDA) challenge, reduce glutamate release from astrocytes, and protect neurons from oxygen-glucose deprivation *in vitro* (26, 27). The balance of these chemokines, and their subsequent activation of microglia may be important (20).

### Blood-brain barrier dysfunction

An influx of peripheral immune cells escalates the CNS host response to *M. tuberculosis*. Infiltration into the CNS is aided by increased BBB permeability in TBM. Several mediators of BBB dysfunction are implicated including matrix metalloproteases (MMP), vascular endothelial growth factor (VEGF), caspases, and cytokines (IFNγ, IL-1β, TNF, IL-8, IL-6), but the mechanisms of dysfunction are not fully understood (28).

#### Matrix metalloproteases

MMP are zinc-containing proteases which degrade extracellular matrix (ECM), including the BBB and are inhibited by tissue inhibitors of metalloproteinases (TIMP). They are critical in tissue homeostasis and development. MMP activate immune mediators and promote leukocyte migration by modulating cytokine and chemokine activity. They are likely to exert a pathological positive feedback role by aiding the influx of neutrophils, monocytes and other leukocytes that promote BBB dysfunction and chemoattraction. This influx may lead to further MMP production, particularly from neutrophils, further driving dysfunction.

MMP-2, also known as gelatinase-A, is the only MMP present in significant amounts in the physiological CNS. *In vitro* models suggest that astrocytes and microglia secrete MMP-1, 3 and 9 in response to interactions with *M. tuberculosis*-infected monocytes, but not upon direct *M. tuberculosis* infection. This MMP expression is driven by cytokines including TNF and IL-1β, although in the case of MMP-2, TNF actually inhibits microglial secretion (29). Inhibition of MMP-3 and 9 results in suppression of inducible nitric oxide synthase (iNOS), IL-1β, IL-1Ra, TNF and IL-6 gene expression in microglia showing that MMP have pro-inflammatory effects on microglia (30).

MMP-9 is a collagenase secreted by astrocytes, microglia and neutrophils which breaks down type IV collagen and downregulates tight junction proteins ZO-1, claudin-5 and occludin between EC in the BBB (31). It is usually undetectable in the normal brain. CSF MMP-9 levels are associated with severity, neurological complications, and brain tissue damage in TBM (32). In addition, compared with aseptic meningitis and non-inflammatory controls, MMP-2 and 9 are raised, and levels remained higher in those TBM patients who developed late neurological complications (33). Adjunctive dexamethasone decreases CSF MMP-9 concentrations but this decline is not associated with improved outcome (34). PLWH who develop TBM-IRIS have higher blood concentrations of MMP-1, 3, 7 and 10 at the time of TBM diagnosis and ART commencement which rise even further at the point of developing TBM-IRIS symptoms (35). However, one study showed no correlation between CSF MMP-9 and outcome in HIV-uninfected TBM patients (36). In a paediatric TBM population, patients with increased CSF MMP-9 levels were twice as likely to have a better outcome (37). Myeloperoxidase, which is mostly expressed in neutrophils, may also contribute to BBB dysregulation by activating MMP-9 (38). Finally, there may be a role for MMP in recovery and remodelling depending on the stage of disease, especially in children (39).

#### Vascular endothelial growth factor

VEGF-A is a potent endothelial growth factor involved in angiogenesis and endothelial permeability. It is a useful biomarker of disease in TBM and is thought to cause BBB dysfunction through its effects on the ECM and tight junctions. Plasma and CSF VEGF-A concentrations are significantly higher in TBM patients than controls although not associated with outcome (40). In a study looking for a CSF biosignature for TBM using proteomics in HIV-uninfected children, a combination of VEGF-A, IFNγ and myeloperoxidase had a sensitivity and specificity of 91.3% and 100% respectively compared to a heterogeneous non-TBM group, suggesting VEGF-A as an important contributor in TBM pathogenesis (41). Studies have also shown that VEGF-A increases MMP-9 activation (42).

#### Adhesion molecules

Adhesion molecules intercellular adhesion molecule 1 (ICAM-1), and vascular cell adhesion molecule 1 (VCAM-1), P-selectin and E-selectin also play a role in BBB dysregulation and leukocyte transmigration and supernatant concentrations increased 25-, 54-, 44-, and 53-fold respectively in an *M. tuberculosis*-stimulated BBB *in vitro* model compared to controls in association with increased leukocyte and neutrophil transmigration (31). Manyelo et al. also reported a second biomarker combination which performed equally well in discriminating TBM and non-TBM, which included soluble ICAM-1, myeloperoxidase, CXCL8 (IL-8), and IFNγ (41), suggesting a key role for ICAM-1 in TBM pathology.

#### Pericytes

Whilst pericytes are known to be critical to the integrity of the BBB, little is understood about their role in BBB dysfunction in TBM. Pericytes cover up to 70% of the CNS surface of the BBB. A recent study used a human cerebral endothelial barrier model to show that paracrine cytokine signalling from monocyte-derived macrophages (MDM) acted on pericytes to enhance neutrophil transmigration via production of a repertoire of neutrophil chemokines (43), suggesting pericytes may be important in neutrophil CNS infiltration in TBM. They used MDM to model perivascular macrophages embedded in the BBB. The authors propose inhibition of atypical chemokine receptor 1 (ACKR1), which transcytoses C-C and C-X-C chemokines across the endothelium, to block the effect of pericyte-derived chemokines on neutrophils, rather than targeting the broad range of individual chemokines. The contribution of pericyte signalling to immunopathology in TBM is unknown, but such therapies may be a focused tool to control neutrophil influx.

#### Other CNS barriers

Other CNS barriers including the ventricular and meningeal BCSFB, and the arachnoid barrier, have not been investigated in TBM, but may be important in pathology. In fact, one study which infected mice with pulmonary *M. tuberculosis* and examined their choroid plexus and BBB using electron microscopy at post-mortem found disruption of the BCSFB, but not the BBB (44).

The inflammatory response to lipopolysaccharide (LPS) and several bacteria, but not *M. tuberculosis*, has been studied using gene expression analysis in *in vivo* and *in vitro* models of the choroid plexus. LPS exposure is associated with increased expression of adhesion molecules, chemokines, and inflammatory miRNAs (19). *Streptococcus suis* disrupts the integrity of epithelial choroid plexus cells by downregulating tight junction proteins (TJP), increases adhesion molecule expression and demonstrates transepithelial migration within polymorphonuclear leukocytes (19, 45). Whether *M. tuberculosis* has a similar effect on the BCFSB remains to be explored.

#### Conclusions

In conclusion, signalling networks between infected microglia, astrocytes, endothelial cells, perivascular macrophages and pericytes lead to the release of cytokines, VEGF-A, MMP, and increased expression of surface adhesion molecules, leading to BBB dysfunction, chemotaxis and an influx of leukocytes into the CNS. These cellular interactions in TBM are poorly understood, as is the balance of their inflammatory mediators between pathology and protection and warrants further investigation. In addition, similar mechanisms of dysfunction have been seen at the choroid plexus in other bacteria but have not been investigated in *M. tuberculosis*. The roles of the meningeal BCSFB and arachnoid barrier in TBM immunopathology are unknown.

### The innate response

#### Inflammasome activation

The role of the inflammasome has become increasingly studied in TBM and other forms of tuberculosis in recent years. Microglial, macrophage and DC TLR recognition of *M. tuberculosis* triggers the inflammasome pathway leading to pyroptosis and the activation of pro-inflammatory IL-1β and IL-18. Inflammasomes are divided into canonical and non-canonical. The canonical inflammasomes involve the effector caspase 1 and include NLRP3 (NOD-, LRR- and pyrin domain-containing protein 3), AIM2 and NLRC4. NLRP3 is triggered by various damage-associated molecular patterns (DAMP), such as as adenosine triphosphate (ATP) from dying cells, and pathogen-associated molecular patterns (PAMP) including those found on *M. tuberculosis*, whereas AIM2 and NLRC4 are triggered by intracellular nucleic acids and Gram-negative secretion systems respectively (46).

The non-canonical inflammasome was first described in 2011 and is characterised by a caspase 4/5-dependent pathway in humans, and caspase 11 in mice. It was initially thought to be triggered by LPS only, which is not found in *M. tuberculosis*, but oxidized 1-palmitoyl-2-arachidonoyl-sn-glycero-3-phosphorylcholine (oxPAPC), a DAMP released by dying cells, and the cyclic GMP-AMP synthase/stimulator of IFN genes (cGAS/STING) pathway, which recognises intracellular bacteria have now also been implicated (47). The different roles of caspases 4 and 5 remain poorly understood.

In TBM, a study comparing blood bulk RNA-Seq in paediatric TBM patients with healthy controls found significant upregulation of transcripts associated with PRR antigen recognition, inflammasome activation and IL-1 signalling (32). Another study compared adult HIV-associated TBM patients with and without immune reconstitution inflammatory syndrome (IRIS) and found enrichment of transcripts associated with canonical and non-canonical inflammasome activation in those with IRIS following anti-tubercular treatment (ATT) as well as significantly higher caspase 1 concentrations in the CSF (48).

Whilst inflammasome activation is associated with IRIS, it is likely that the inflammasome plays a protective as well as a detrimental role in TBM. Programmed cell death (PCD) kills *M. tuberculosis* along with the host cell, and *M. tuberculosis* inhibits NLRP3 inflammasome activation via PknF and Hip1, and the AIM2 inflammasome via an ESX-dependent mechanism in an effort to favour necrosis (49). However, lymphocyte-derived IFNγ inhibits the NLRP3 inflammasome in mice mediated by nitric oxide (NO) implying NO produced by the adaptive immunity modulates the innate inflammatory response (50).

There is conflicting evidence as to whether IL-1β plays a protective or detrimental role in TBM and a fine balance may be required. IL-1β is essential for the immune response against TB. IL-1 has been shown to confer host resistance by limiting excess type 1 IFN production via the induction of eicosanoids and helps regulate the containment and replication of bacilli (51). In an Indonesian HIV-uninfected TBM study, CSF IL-1β was elevated 44-fold compared to controls but did not predict mortality, nor did levels of IL-6, a downstream target of IL-1β. Interestingly, IL-1Ra, the receptor antagonist of IL-1, was increased 26-fold in TBM and closely correlated with IL-1β levels but did not predict mortality either. In addition, genetic loci known to regulate *IL1B* expression do not associate with mortality from TBM (52).

IL-1β production is not synonymous with canonical inflammasome activation; neutrophil-associated proteases including MMP-2, 3, and 9 can also activate IL-1β and IL-18 independently of the inflammasome (49). Another recently discovered microglial source of IL-1β is the non-canonical caspase-8-dependent inflammasome. Caspase 8 has been found to be important for IL-1β production in microglia (53). The importance of these non-inflammasome sources of IL-1β in TBM are unclear.

#### Neutrophils

There is a clear role for neutrophils in the inflammatory response in TBM. Neutrophils are not found in the brain or CSF in healthy subjects and so neutrophil chemotaxis, infiltration, and activation may play a critical role in the dysregulated immune response in TBM. Once neutrophils have been recruited to the CNS, they phagocytose *M. tuberculosis*. Virulent *M. tuberculosis* initiates necrosis, and the proteases released can damage neighbouring cells and tissues. Macrophages phagocytose necrotic neutrophils and *M. tuberculosis* aiding further *M. tuberculosis* distribution and inflammation (54).

Higher pre-treatment CSF neutrophil counts associate with an increased likelihood of culturing *M. tuberculosis* and neurological events (55), but their association with mortality is variable. In Indonesia, higher CSF and blood neutrophil counts at diagnosis associated with mortality in TBM (56). Significantly increased whole blood transcripts and CSF concentrations of neutrophil-associated proteins including MMP, cathepsin G, lipocalin 2 and human neutrophil peptides 1-3 (α-defensins) are found in TBM-IRIS (48). S100A8, a neutrophil chemotactic protein, is differentially expressed in TBM compared to other forms of meningitis (57), and increased CSF levels of S100A8/9 accompany TBM-IRIS (35). Variations in the *LTA4H* genotype have been shown to predict outcomes in TBM. Leukotriene A_4_ hydrolase (LTA4H) converts LTA_4_ to LTB_4_, a potent neutrophil chemoattractant and pro-inflammatory eicosanoid. Patients with the pro-inflammatory TT genotype of LTA4H have higher CSF concentrations of IL-1β, IL-2 and IL-6 and benefit more from adjunctive corticosteroid therapy (5). A clinical trial stratifying corticosteroid treatment by *LTA4H* genotype in HIV-uninfected TBM patients is ongoing in Vietnam (NCT03100786).

As well as degranulation and phagocytosis, neutrophils activated by *M. tuberculosis* release neutrophil-extracellular traps (NETs), which consist of modified chromatin containing bactericidal proteins such as myeloperoxidase and neutrophil elastase designed to capture and kill bacteria. This release is part of NETosis, a regulated form of cell death, although a ‘vital’ NETosis has now been described allowing cell survival (58). NETs from *M. tuberculosis*-activated neutrophils triggered significant secretion of IL-6, TNF, IL-1β, and IL-10 by macrophages *in vitro* (59). NETs activate platelets and the complement pathway and may promote thrombosis in brain microvessels during TBM. More research is needed to understand the role of NETosis in TBM.

In summary, whilst not found in the healthy CNS, neutrophils play a central role in TBM immunopathology with neutrophil-associated proteins and transcripts characterising more severe phenotypes. Further research is required to understand the exact mechanism of this damage. Studies are limited by neutrophil loss *ex vivo* between the bedside and laboratory which can lead to bias in transcriptomic studies. In addition, the role of NETosis in inflammation and thrombosis in TBM deserves further attention.

#### Cytokine release

**Figure 3:**
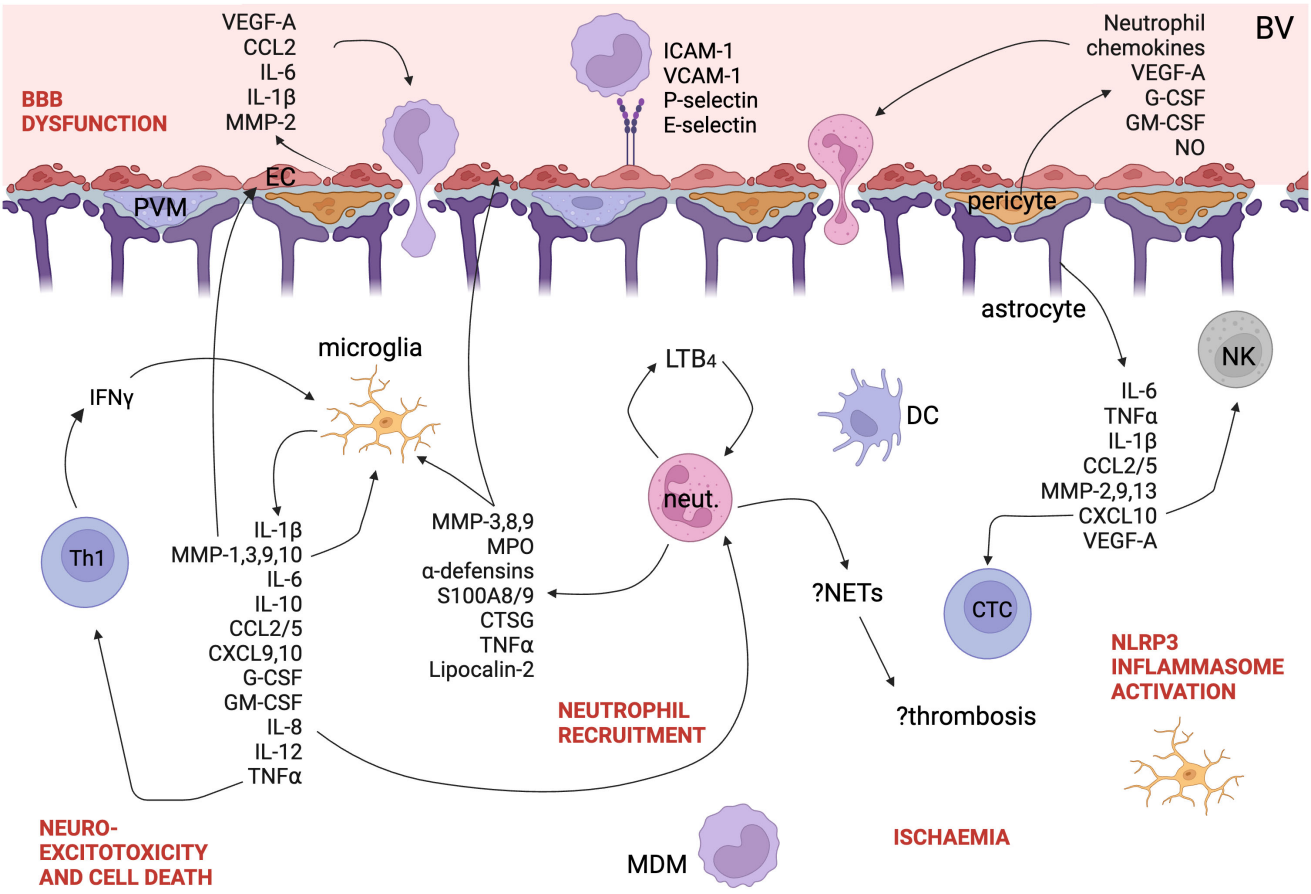
A schematic of the immune landscape of TBM. Cytokines, chemokines, and other proteins implicated in the pathogenesis of TBM are shown. Polymorphisms down- and up-regulating LT4_B_ pathway are both associated with worse outcomes in TBM. Created with BioRender.com. EC, endothelial cell; DC, dendritic cell; MDM, monocyte-derived macrophage; Th1, Th1-helper cell; NETs, neutrophil extracellular traps; neut., neutrophil; LT4_B_, leukotriene 4_B_; NLRP3, NOD-, LRR- and pyrin domain-containing protein 3; PVM, perivascular macrophage; BBB, blood-brain barrier; BV, blood vessel; NK, natural killer cell; NETs, neutrophil extracellular traps; CTL, cytotoxic T lymphocyte; VEGF, vascular endothelial growth factor; CCL, C-C motif chemokine ligand; IL, interleukin; MMP, matrix metalloprotease; ICAM, intercellular adhesion molecule; VCAM, vascular cell adhesion molecule; LTB4, leukotriene B4; IFN, interferon; CXCL, C-X-C motif chemokine ligand; G-CSF, granulocyte colony-stimulating factor; GM-CSF, granulocyte-macrophage colony-stimulating factor; TNF, tumour necrosis factor; CTSG, cathepsin G; MPO, myeloperoxidase; NLRP3, NACHT, LRR, and PYD domains-containing protein 3; NO, nitric oxide.

As discussed above, the activation of microglia and the influx of leukocytes into the CNS in TBM results in cytokine release (**Figure 3**). Cytokines and their expression in cells can be easily measured in blood and CSF and have been well studied in TBM but for many cytokines, it is clear that labelling them protective or pathogenic is simplistic and they can play both roles depending on the cell, the disease course, the patient and the pathogen. In contrast to the hypothesis that death in TBM is due to hyperinflammation, in HIV-uninfected adults in Vietnam, patients who died had an attenuated inflammatory response with lower CSF cytokine concentrations (55). A systematic review of cytokine levels in lumbar CSF in TBM found that IFNγ, IL-13, and sIL-2R were increased in TBM compared with other causes of bacterial meningitis, whereas TNF, IL-1β, IL-1Ra, IL-8, IFNγ, sIL-2R, IL-13, and IL-17 were increased compared to viral or aseptic meningitis (60). Kwon et al. compared 10 pre-treatment definite and probable TBM patients with 45 patients with other neurological infections and found that CSF IL-12p40, IL-13, MIP-1α, sPD-1, and PD-L1 were significantly increased in TBM (61). sVCAM1, MMP-1, sRAGE, CXCL10 are higher in TBM patients with stroke compared with those without stroke, and other forms of meningitis in children (62).

#### Tumour necrosis factor

TNF is synthesised by several cells found within the inflamed CNS including microglia, macrophages, neutrophils, dendritic cells, lymphocytes, astrocytes and neurons. It has a protective role in the immune response to tuberculosis including granuloma formation and maintenance. TNF^-/-^ mice are highly susceptible to TB dissemination, including to the brain, and early mortality (63). However, TNF is undoubtedly critical in the pathogenesis of TBM. TNF increases the permeability of the BBB, triggers further cytokine release, and correlates with severity in mouse models (64). It is found at high levels at the interface between the cellular and necrotic zones of TB granulomas in post-mortem studies (17). High CSF TNF levels with low IFNγ levels predict TBM-IRIS. Thalidomide, a TNF antagonist, downregulated TNF production and increased survival in a rabbit model of TBM (64). Optimal levels of TNF are likely to be determined by disease course and individual patient factors in TBM.

#### Interferon-gamma

IFNγ is produced by NK cells, and CD8^+^ and CD4^+^ T cells, including γδ cells, and is essential to activate microglia and other macrophages to induce intracellular mycobacterial killing. IFNγ inhibits neutrophil accumulation and survival in *M. tuberculosis*-infected lung both directly and via inhibition of CD4^+^ T cell production of IL-17, which regulates neutrophil recruitment via S100A8/9 production (65, 66). The effect of IFNγ on neutrophil CNS recruitment alongside its macrophage and microglial activity may help explain why low CSF IFNγ concentrations independently predict death in PLWH with TBM (5, 67).

#### Other cytokines

Other important cytokines in TBM include IL-6, IL-12, and IL-10. IL-6 is associated with a more severe presentation of TBM (67). Mice lacking the larger IL-12 subunit, p40, are more susceptible to mycobacterial infections, and inherited IL-12p40 deficiency in humans is associated with an increased risk of TB and failure of PBMC to produce IFNγ. IL-12 secretion by microglia, DC, neutrophils and CNS macrophages induces T helper 1 cell (Th1) differentiation of naïve T cells and stimulates IFNγ and TNF production by NK and T cells. IL-10 is produced by microglia upon TLR stimulation as well as neurons and has anti-inflammatory activity. IL-13 is produced by T cells and also has anti-inflammatory properties. Despite being significantly raised in TBM compared with bacterial, viral and aseptic meningitis (60), there is no literature investigating the role of IL-13 in TBM. Lower levels in bacterial meningitis could be explained by a neutrophil predominance. Given that IL-13 inhibits macrophage inflammatory cytokines and Th1 cells, an imbalance may contribute towards immunopathology in TBM.

#### Pro-inflammatory eicosanoids and specialised pro-resolving mediators

The role of specialised pro-resolving mediators (SPM) and pro-inflammatory eicosanoids has been investigated in TBM (68). SPM are a recently discovered genus of lipid mediators which terminate inflammation and promote tissue repair and include lipoxins, resolvins, protectins and maresins. They can counter regulate the production of pro-inflammatory cytokines and eicosanoids, control leukocyte trafficking and phenotype and regulate the phagocytosis and killing of bacteria. Using metabolomics, Colas et al. found that pre-treatment concentrations of SPM and increased levels of pro-inflammatory eicosanoids including prostaglandins and cysteinyl leukotrienes in lumbar CSF significantly associated with mortality and higher British medical research council (BMRC) grades. Fatty acid substrates of SPM and enzyme activity were not reduced in more severe cases, and CSF white cell counts were similar implying that reduced SPM concentrations were due to differences in cell phenotype or population rather than absolute leukocyte numbers (68). It is known that different macrophages have distinct lipid mediator profiles (69) and further understanding of the cell populations responsible for the balance of SPM and eicosanoids may provide opportunities for novel HDT in TBM. Aspirin plays a role in the balance of these mediators in TBM; Mai et al. showed dose-dependent inhibition of thromboxane B_2_, the inactive metabolite of the rapidly degraded pro-inflammatory eicosanoid thromboxane A_2_, and upregulation of protectins in lumbar CSF in a study comparing 81mg and 1000mg aspirin daily with placebo. Thromboxane B_2_ levels were significantly lower in lumbar CSF at day 30 in the aspirin, 1000mg group compared to placebo (70). Phase 3 clinical trials investigating aspirin in TBM are ongoing.

### The adaptive response

#### T cells

The role of the adaptive immune response against TBM is well documented. CD137L expression by resident microglia activates infiltrating T cells. In addition, infiltrating neutrophils promote the activation of CD4^+^ T cells via DC. Whilst certain T cell phenotypes may contribute to excess inflammation, their positive role in reducing morbidity and mortality from TBM is clearer than other leukocytes such as neutrophils. TBM is characterised by a T cell-dominant lymphocytic CSF. Th1 cells are essential to activate macrophages and microglia via IFNγ, and a CSF IFNγ-release assay (IGRA) response is seen in TBM (71). In an Indonesian cohort of 160 HIV-uninfected TBM patients, there were reduced αβ cells, γδ cells, NK cells and MAIT cells in blood, but increased neutrophils and classical monocytes compared with pulmonary TB (PTB) and healthy controls. The T cell make-up of the CSF was 57% αβ, 13% NK, 1.5% γδ, 0.4% MAIT, 0.06% NKT revealing that important T cell subsets are found in the CSF of TBM and may play an unknown role in the host immune response. Higher levels of CSF αβ cells and NK cells were associated with better outcomes in TBM (72). Effector CD8^+^ and Vγ9/Vδ2 T cells have also been implicated in TBM (73, 74).

Although a robust T cell response is important, so is the nature of the response. *Strongyloides stercoralis* co-infection is significantly associated with reduced pro-inflammatory cytokine concentrations in the CSF before treatment and reduced neurological complications at three months in adult TBM. The Th2 immune response induced by *Strongyloides stercoralis* may non-specifically inhibit the pro-inflammatory Th1 response and protect the host from immunopathology (75).

#### T cell functional impairment

Impaired T cell function is a general feature of TB which is also seen in TBM (76). A study examining blood bulk RNA transcripts of HIV-uninfected children with TBM found that T cell development and function were downregulated, along with a reduced *in vitro* T cell response to non-specific mitogen activation, which resolved with treatment (77). In addition, children with TBM have downregulated T cell activation and signalling in whole blood transcripts compared with healthy controls, but not in ventricular CSF versus other neurological infections (32). In a Korean study, soluble programmed death protein 1 (sPD-1) and programmed death ligand 1 (PD-L1) were increased in lumbar CSF in probable or definite TBM compared with other inflammatory or infectious neurological conditions (61). PD-1 is expressed on the cell surface and limits the activation and function of T cells. PD-L1 blocking increases CD8 T cell cytotoxicity against *M. tuberculosis*-infected macrophages, especially M1-polarised (78). However, anti-PD-1 monoclonal antibodies such as pembrolizumab also predispose to TB reactivation (79), and efforts to resolve impaired T cell function with PD-1/PD-L1 blockage should be approached with caution.

#### MicroRNAs

MicroRNAs (miRNAs) are highly conserved non-coding RNAs involved in the regulation of gene expression. The miR-29 family regulates the adaptive immune response, including T cell differentiation (80), and suppress IFNγ production by directly targeting IFNγ mRNA. In a mouse model with transgenic expression of a target which competes with endogenous miR-29 targets thereby reducing the effect of miR-29 expression, a greater IFNγ and Th1 response and lower *Listeria monocytogenes* burden were seen in infected mice suggesting miR-29 suppresses the immune response to intracellular bacteria. One study found that mi-R29a levels were increased in PBMC and lumbar CSF of children with TBM compared with healthy controls and were associated with markers of severity (81). Increased levels of mi-R29a may impair the adaptive response to TBM and mi-R29 could be a promising therapeutic target.

Overall, T cells are essential for a robust host response to TBM, and impaired T cell function may contribute to worse outcomes. However, characterising the role of specific T cell subsets such as MAIT, γδ and NKT cells has been limited by low CSF cell numbers, and the granularity of bulk RNA sequencing. The polarisation of T cells is likely to be important, as well as epigenetic influences.

#### B cells

There is very limited data on B cells in TBM. B cells are found in the CSF in health and TBM disease. B cells are necessary for granuloma formation and in active TB, frequencies are lower and B cells appear to be functionally impaired (82). In addition to antibody production, B cells produce cytokines and modulate the function of T cells, macrophages and neutrophils. Functional impairment of B cells may therefore have consequences for T cell activation (83), but how this may influence CNS infection is unknown.

### Neuroexcitotoxicity

#### The glutamate-glutamine-GABA cycle

**Figure 4:**
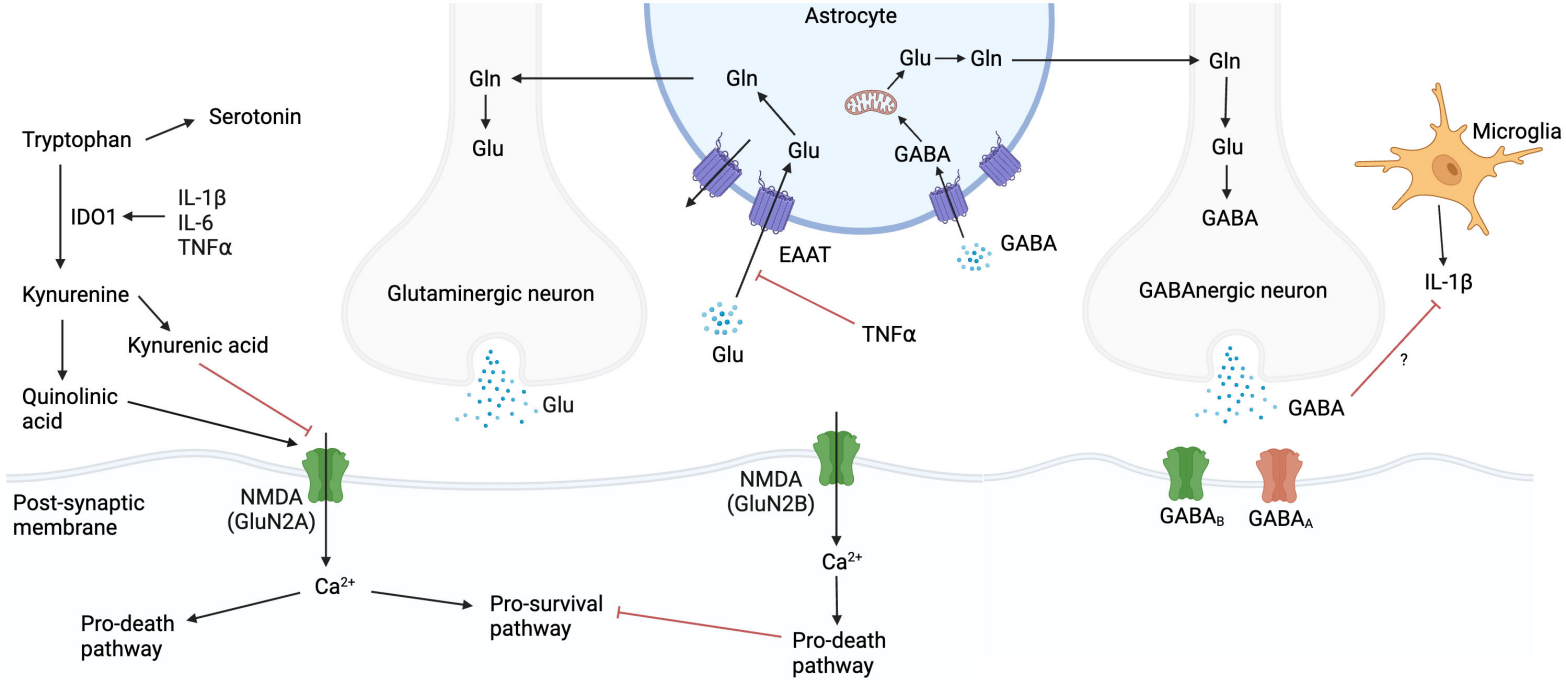
Glutamate, glutamine, and GABA cycle. Glutamate concentrations are tightly controlled, and ambient levels are maintained mainly by astrocytes. Prolonged glutamate exposure leads to neuronal damage, which is exacerbated under conditions of oxidative or metabolic stress. Tryptophan can be metabolised via the serotonin or kynurenine pathways. IL-1β, IL-6 and TNF upregulate IDO1 which favours the kynurenine pathway. Kynurenine can be metabolised to either quinolinic acid or kynurenic acid, which agonise or antagonise NMDA receptors respectively. Created with BioRender.com. Gln, glutamine; Glu, glutamate; GABA, γ-aminobutyric acid; IL, interleukin; NMDA, N-methyl-D-aspartate; TNF, tumour necrosis factor; Ca^2+^, calcium ion; IDO1, indoleamine 2,3-dioxygenase.

Glutamate is a neuroexcitatory neurotransmitter, regulated by the glutamate-glutamine cycle, which acts on post-synaptic neurons, oligodendrocytes, glial cells, and astrocytes (84) (**Figure 4**). Glutamate concentrations are tightly controlled, and ambient levels are maintained mainly by astrocytes (85). Prolonged glutamate exposure leads to excitotoxic neuronal damage, which is exacerbated under conditions of oxidative or metabolic stress e.g., ischaemia (86). The balance between glutamate and γ-aminobutyric acid (GABA), a neuroinhibitory neurotransmitter, is crucial for neurologic homeostasis (87). In addition, glutamate and GABA influence, and are influenced by, CNS immunity. TNF inhibits glutamate re-uptake by astrocytes, potentially increasing ambient glutamate concentrations in the extracellular space and worsening excitotoxicity (88, 89). GABA has been shown to inhibit IL-1β production in macrophages (90), and this effect may occur in microglia. In addition, EC express NMDA receptors, and glutamate induced endothelial permeability in a rat model has been demonstrated via a NO and cyclic guanosine monophosphate (cGMP) signalling cascade which rearranged TJP away from cell-cell contact regions (91, 92).

Glutamate-driven neuro-excitotoxicity underlies pathogenesis in a wide range of neurological conditions including Alzheimer’s disease and traumatic brain injury (93, 94). In the ventricular CSF of HIV-uninfected children with TBM, transcripts associated with glutamate release, post-synaptic NMDA receptor binding and calcium influx were enriched, suggesting a role for glutamate in TBM-associated neuro-excitotoxicity (32). A study in South Africa used hydrogen nuclear magnetic resonance (^1^H NMR) metabolomics to compare 18 metabolites in the lumbar CSF of HIV-uninfected paediatric TBM with non-meningitis controls and found glutamate and glutamine significantly elevated. The authors hypothesised that transporter function is reduced in both astrocytes and neurons in TBM (95).

Caution must be used when interpreting glutamate levels. HIV-1 affects glutamate concentrations in the brain and in studies of PLWH appropriate controls must be used (89). In addition, glutamate is actively cleared from the CSF both into the blood via choroid plexus epithelial cells, and into the brain via ependymal cells and so lumbar CSF may be a poor proxy for extracellular brain concentrations (96). In addition, whether glutamate is degraded as it descends the spinal tract is unknown. Magnetic resonance spectroscopy (MRS) is an established non-invasive method for direct detection of brain metabolites. Glutamate and GABA are amongst those metabolites detectable (97, 98) and MRS could allow the investigation of neurotransmitter concentrations *in vivo*, although it cannot always distinguish between glutamate and glutamine, or extracellular and intracellular levels, concentrations being about, 1000-fold higher in the latter.

#### The tryptophan pathway

The tryptophan pathway has been implicated in TBM pathology in recent years. Tryptophan, an essential amino acid, can be metabolised via the serotonin or kynurenine pathways. IL-1β, IL-6, IFNγ and TNF upregulate indoleamine 2,3-dioxygenase (IDO1) which induces the kynurenine pathway (99, 100). Kynurenine pathway metabolites have broad antibacterial properties although this is unlikely to be significant in TB infection (101). IDO1 may inhibit IL-17 expression and subsequent neutrophil migration in PTB (100). Blockage of IDO1 activity reduces the clinical manifestations of TB in macaques (102). The tryptophan pathway may play a role in neuroexcitotoxicity (**Figure 4**). Kynurenine can be metabolised to either quinolinic acid or kynurenic acid, which agonise or antagonise NMDA receptors respectively. Therefore, over induction of the quinolinic acid pathway may contribute to excitotoxicity. Higher lumbar CSF tryptophan levels correlate positively with mortality and negatively with CSF IFNγ in TBM (103, 104). In addition, CSF kynurenine metabolites correlate with CSF inflammation and markers of blood-CSF leakage, in both HIV-uninfected and PLWH. Induction of the kynurenine pathway may benefit the host by reducing the amino acid energy source for *M. tuberculosis* and triggering a kynurenine-induced macrophage response (103). In the CNS, IDO1 expression has been shown in macrophages, DC, microglia and choroid plexus epithelial cells (105, 106). The relative contributions of these cells to tryptophan homeostasis in TBM and the compartmental differences in concentrations of tryptophan and its metabolites are unclear.

The role of neurotransmitters in host damage and immunity in TBM is an exciting area. NMDA receptor antagonists have been unsuccessful in improving stroke outcomes due to poor tolerance at therapeutic doses, possibly because of their effects on pro-survival signalling in neurons. However, downstream inhibitors specifically targeting NMDA receptor pro-death pathways could be further investigated in TBM and are an area of active research in stroke (107).

### Host genetic immune factors

Host genetic polymorphisms which associate with the risk of developing or dying from TBM can give us clues to the host immune response (**Table 1**). As mentioned above, a single nucleotide polymorphism (SNP) in the *LTA4H* gene has been associated with inflammatory cell recruitment, severe IRIS, survival and response to adjunctive corticosteroids which was reproduced in a large Vietnamese HIV-uninfected cohort (5, 119). This association was not seen however in an Indonesian study (56), but nonetheless may herald an era of more personalised therapy. Other polymorphisms have been associated with developing TBM include those encoding for mannose-binding lectin (*MBL2*), TLR2, TLR9, toll-like adaptor protein (*TIRAP*), toll-like receptor/IL-1R signalling (*PKP3-SIGIRR-TMEM16J* gene region), vitamin D receptor (*VDR*) and the lung protein mucin-5AC (*MUC5AC*) (108, 109, 111–116). Worse outcomes in TBM have been associated with polymorphisms in *SPN* which encodes for CD43, a surface glycoprotein found on immune cells involved in *M. tuberculosis* adhesion and pro-inflammatory cytokine release (117). These polymorphisms are mostly in genes responsible for the innate immune response.

**Table 1:** Polymorphisms associated with susceptibility to outcome in TBM.

Gene	Function	Sample size			Location	Polymorphism	Association	Reference
		TBM	PTB	Cont.				
LTA4H	Leukotriene A_4_ hydrolase; an enzyme which converts LTA_4_ to LTB_4_, a neutrophil and macrophage chemoattractant and pro-inflammatory eicosanoid	764	NA	NA	Vietnam	rs17525495 C>T	Increased mortality in HIV-uninfected patients with CC vs. TT genotype (HR 3.4, 95% CI: 1.05-11.0) in TBM treated with corticosteroids; higher concentrations of IL-1β, IL-2 and IL-6 expression in TT genotype	(5)
		608	NA	NA	Indonesia	rs17525495 C>T	No significant difference in survival between genotypes	(56)
MBL2	Mannose-binding lectin; binds and opsonises *M. tuberculosis* facilitating uptake	33	102	243	South Africa	Gly54Asp allele	Heterozygous/homozygous mutants associated with protection from TBM (OR of WT vs. mutant for developing TBM 4.69, 95% CI: 1.64-13.41)	(108)
TLR2	PRR mediating recognition of *M. tuberculosis*	130	130	130	India	Arg753Gln and Pro631His	No association	(109)
		111	230	386	China	rs3804099 (597T>C)	Associated with PTB but not TBM	(110)
		175	183	389	Vietnam	rs3804099 (597T>C)	CC mutant genotype increases risk of developing TBM (OR 5.28, 95% CI: 2.20-12.65) as well as miliary TB. Association increases with increased neurological symptoms	(111)
TLR9	PRR mediating recognition of *M. tuberculosis*	281	355	758	Vietnam	rs352142	Increased susceptibility to TBM (OR 2.36, 95% CI: 1.43-3.87) but no association with mortality. n=352 TB (discovery), n=339 TB (validation)	(112)
TIRAP	Toll adaptor protein	175	183	392	Vietnam	558C>T allele	SNP associated with increased susceptibility to TBM (OR 3.02, 95% CI: 1.79-5.09)	(113)
		10	44	16	South Africa	558C>T allele	CT heterozygotes associated with increased risk of TBM vs. other TB and controls in mixed ancestry group	(114)
PKP3-SIGIRR-TMEM16J region	Negative regulator of TLR/IL-1R signalling	297	394	758	Vietnam	rs10902158 rs7105848 rs7111432	Increased susceptibility to TBM and PTB; OR 1.53, 95% CI 1.19-1.97 (rs10902158), OR 1.52, 95% CI: 1.18-1.96 (rs7105848), OR 1.61, 95% CI: 1.2-2.17 (rs7111432). n=170 TBM (discovery), n=127 TBM (validation)	(115)
VDR	Vitamin D receptor	130	130	130	India	rs731236 (Taq1 VDR gene) and rs7975232 (Apa1 VDR gene)	Both genes associated with TBM susceptibility: OR 3.53, 95% CI: 1.95-6.40; CC – OR 5.97, 95% CI: 1.89-18.84 (rs731236); TG OR 2.86, 95% CI: 1.58-5.17 (rs7975232)	(109)
MUC5AC	Lung mucin	1172	NA	1139	Vietnam/ Indonesia	rs28737416 C>T allele	TT higher susceptibility to TBM (OR 1.24, 95% CI 1.03-1.49) but not PTB; higher mortality in TT & TC in a Vietnamese discovery, and Vietnamese and Indonesian validation cohorts; lower CSF TNF and IFNγ concentrations in T allele. n=407 TBM (susceptibility cohort), n=210 TBM (Vietnam discovery), n=87 TBM (Vietnam validation), n=468 TBM (Indonesia validation)	(116)
SPN	Surface glycoprotein (CD43) involved in *M. tuberculosis* adhesion and cytokine induction	297	394	758	Vietnam	rs17842268 rs12596308 rs4788172	All three SNP associated with increased susceptibility to TBM (rs4788172, OR 1.64, 95% CI: 1.04–2.59; rs17842268, OR 2.20; 95% CI: 1.29–3.76; rs12596308, OR 2.38, 95% CI: 1.47–3.89). Associations stronger than PTB. rs17842268 associated with higher mortality in TBM (HR 2.7, 95% CI: 1.1-6.5). rs17842268 and rs12596308 associated with focal deficit at TBM presentation.	(117)
CCL2/ MCP-1	Chemokine regulating migration and infiltration of monocytes and several other immune cells	118	105	338	China	rs4586 C>T	TT genotype associated with lower CSF mononuclear WCC in TBM vs. CC/TC (p=0.001). Study also included 78 non-TBM EPTB	(118)

LTA4H, leukotriene A4 hydrolase; MBL2, mannose-binding lectin 2; TLR2, toll-like receptor 2; TLR9, toll-like receptor 9; TIRAP, Toll-interleukin-1 Receptor (TIR) domain-containing adaptor protein; VDR, vitamin D receptor; MUC5AC, mucin 5AC; SPN, sialophorin; CCL2, C-C Motif Chemokine Ligand 2; MCP-1, monocyte chemoattractant protein 1; IL, interleukin; PTB, pulmonary tuberculosis; EPTB, extrapulmonary tuberculosis; TBM, tuberculous meningitis; OR, odds ratio; HR, hazard ratio; SNP, single nucleotide polymorphism; NR, not reported; PRR, pattern recognition receptor; Cont., ethnically matched healthy controls with no features of active TB; NA, not applicable.

Even within the same geographical region, identifying polymorphisms can be limited by genetic differences. This was demonstrated recently in a genome-wide association study (GWAS) of South African children with TBM versus healthy controls and PTB cases which did not find any associations with TBM susceptibility (120). The authors attribute this to the sample size, age differences and the ethnic heterogeneity of the study population.

### Differences in the host immune response in HIV-1 infection

Only a few studies have directly compared HIV-infected and HIV-uninfected patients in TBM. TBM in the context of HIV-1 infection has a distinct pathophysiology. HIV-1 is a risk factor for PTB, extrapulmonary TB, bacillaemia and consequently TBM. Poorer outcomes are seen in PLWH, who often present with occult disease and up to 50% can develop TBM-IRIS depending upon timing of ART. Although this population is the most immunosuppressed, the potential for immunomodulation with HDT is greatest given the higher mortality and more dysregulated immune response. A comprehensive understanding of the host immune response in PLWH is therefore paramount to developing novel treatment options.

HIV-1-associated TBM is characterised by increased bacillary load and CNS neutrophil counts. Mean CSF neutrophils and cytokines are higher in HIV-1 co-infection, especially in those with CD4 counts less than 150 cells/mm^3^ in whom increased death is also seen (5). In a review of cytokines in TBM, IL-1β, TNF, and IL-2 were increased in PLWH compared with HIV-uninfected patients (60). Higher neutrophils are associated with an increased likelihood of culturing *M. tuberculosis* in PLWH (55). As well as an impaired immune response, disruption of the BBB by HIV-1 may aid *M. tuberculosis* entry into the CNS and subsequent leukocyte infiltration in TBM through the HIV-1 proteins gp120, trans-activator of transcription (Tat), negative regulatory factor (Nef) and viral protein R (Vpr). These proteins also induce MMP production, cytokine release, chemotaxis, astrocyte disturbance and TJP downregulation (121).

The pathogenesis of IRIS is driven by *M. tuberculosis* load and may result from excessive neutrophil-mediated inflammation. High CSF neutrophil counts and *M. tuberculosis* culture positivity at presentation predict TBM-IRIS, as well as a combination of high CSF TNF and low IFNγ. Neutrophil mediators, in particular IL-17-induced S100A8/9, are associated with the development of TBM-IRIS. The same longitudinal study revealed that many of the hallmarks of TBM, including MMP, inflammasome markers (IL-1β, IL-18), IL-6 and neutrophils, are increased in TBM-IRIS compared to those who do not develop IRIS, suggesting a spectrum of severity rather than a discrete pathology (35). Another study looking at bulk RNA sequencing changes in the blood of PLWH who did and did not develop TBM-IRIS over time found significantly upregulated neutrophil- and inflammasome-associated transcripts in those who developed TBM-IRIS (48).

## Discussion

This review summarises the existing literature on the human host immune response in TBM. *M. tuberculosis* probably enters the CNS via a combination of transcytosis, paracytosis and immune cell invasion but the various CNS barriers have been poorly studied and modelled. Microglia trigger the initial inflammatory response by attracting, stimulating, and activating other leukocytes, which is followed by CNS barrier dysfunction mediated by cytokines, MMP, VEGF, adhesion molecules and pericytes. The inflammasome plays a key role in the innate response and increased CSF concentrations are associated with TBM and TBM-IRIS, but identifying HDT targets requires further understanding of the mechanisms of inflammasome activation in TBM. Neutrophils and their associated proteins are associated with poorer outcomes and TBM-IRIS. The contribution of NETosis is unknown. The balance of specialised pro-resolving mediators and pro-inflammatory eicosanoids is associated with mortality and controlling this balance may be a promising target for HDT beyond aspirin in the future. T cells are vital for the response to *M. tuberculosis* and impaired function is seen in TBM. MiR-29, a microRNA mediator of T cell differentiation and IFNγ production, associates with poorer outcomes. Glutamate concentrations are increased in lumbar CSF and targeting the glutamate-GABA cycle and tryptophan pathway may reduce neuro-excitotoxicity in TBM.

### Potential future host-directed therapies

Host-directed therapy can be divided into drugs which dampen or augment host immunity. Given that excess inflammation drives morbidity in TBM, HDT is believed to be a necessary adjunct to ATT to dampen the immune response. Corticosteroids reduce acute mortality in HIV-uninfected patients (122), but despite these, mortality in TBM remains unacceptably high. In addition, a recent study investigating corticosteroids in TBM patients with advanced HIV found only a trend towards reduced mortality in those who received dexamethasone which did not reach significance, with similar incidences of neurologic disability and new neurologic events (6). This study throws into question the routine use of steroids in PLWH, potentially leaving no evidence-based adjuncts in this group. A better understanding of TBM would allow us to develop more selective HDT and improve outcomes.

Anti-TNF drugs may have a role in TBM but when to initiate them and in whom are important questions that need answering. The only randomised controlled trial (RCT) of an anti-TNF agent was high-dose thalidomide in a paediatric TBM population, which was stopped early due to adverse events (123). Despite that, there have been promising results with the use of infliximab and lower-dose thalidomide in paediatric and adult TBM in paradoxical reactions refractory to steroids (124, 125). A retrospective cohort study showed improved outcomes with up to three doses of 10mg/kg infliximab in patients with symptomatic tuberculomas, spinal cord involvement with paraparesis and optochiasmic arachnoiditis despite adequate ATT and corticosteroid therapy. The median duration from start of ATT and corticosteroid treatment was six months. The cohort only included one PLWH (126). A randomised trial is needed to investigate whether these improved outcomes with infliximab are seen more generally when administered alongside ATT from diagnosis.

In a meta-analysis of three RCT, aspirin has been shown to reduce new-onset stroke in TBM (HR 0.51, 95% CI: 0.29-0.87) but not mortality (127). Low-dose aspirin has anti-platelet activity alone, whereas high-dose aspirin has additional anti-inflammatory properties by inhibiting pro-inflammatory prostaglandins and thromboxane A_2_. Only two RCT included high-dose alongside low-dose aspirin, at different doses (1000mg/day and 100mg/kg/day) and in different populations (adults and children) (70, 128). Since the meta-analysis, an additional phase 2A study including high-dose aspirin at 1000mg/day demonstrated good safety (129). Existing studies in high-dose aspirin are underpowered to detect improvements in mortality but phase 3 trials are ongoing in adults (NCT04145258) and children (ISRCTN40829906) which may help to establish whether aspirin should be given routinely in TBM, and if so, at what dose.

Promising future HDT could include IDO1 blockers to downregulate the kynurenine pathway, NMDA receptor inhibitors, endothelial ACKR1 inhibitors to block chemokine transcytosis, anthracyclines as NETosis suppressing drugs (130), and small molecular inhibitors of the NLRP3 inflammasome pathway e.g., MCC950, glyburide, and sulforaphane (131), but these will require further research both *in vitro* and in a rabbit model. Ongoing studies of HDT in PTB include statins, tyrosine kinase inhibitors, metformin, phosphodiesterase inhibitors, and NSAIDS/COX2 inhibitors (132). These may be applicable to TBM and should be monitored closely.

### Ongoing challenges in TBM research

Our understanding of the immunopathology in TBM remains limited and hampers the development of HDT. Several limitations to existing data, and future challenges in TBM research exist. In a small brain microdialysis study in paediatric TBM, brain extracellular fluid (ECF) cytokine levels were lower than ventricular CSF, which were in turn lower than lumbar CSF (133). In addition, Rohlwink et al. have demonstrated a compartmentalised response, with differences between blood, lumbar CSF, and ventricular CSF (32), all of which are likely to be proxies of varying accuracy of the immune response occurring primarily in the basal cisterns, brain leptomeninges and subpial border zone. Heterogeneities between PLWH and HIV-uninfected patients, and adults and children limit extrapolation. Existing data have come from bulk transcriptomics and proteomics, and the role of individual immune cell subsets remains unclear. The difficulty acquiring CSF samples has led to a reliance on blood which may be influenced by TB elsewhere, or other systemic confounders. The universal use of corticosteroids in the treatment of TBM, given for the very purpose of moderating the immune response, influences any data in a way that is poorly understood, and makes it challenging to find suitable controls. The poor sensitivity of diagnostic tools means relying on clinical criteria or missing the majority of cases. The Marais criteria classify TBM patient as ‘possible’, ‘probable’ or ‘definite’ (134). Patients classified as ‘possible’ will inevitably include non-TBM cases; sensitivity can be adjusted for improved specificity by excluding ‘possible’ cases, which can mean lower enrolment in clinical trials, especially in settings of high HIV-1 prevalence. Finally, a lack of *in vivo* and *in vitro* brain/BBB models, as well as containment level 3 (CL3) considerations, hinder pre-clinical research.

### Research gaps and the path forward

There has been notable progress in TBM research in recent years. Historical challenges have been partially overcome by recent developments in multiomics technologies. It is increasingly evident that host immunity goes beyond the recognised elements of the immune system as shown by the complex interactions of the neurovascular unit at the BBB, and the influence of the tryptophan pathway and glutamate-glutamine-GABA cycle. At a cellular and molecular level, areas of future research could include microglial and macrophage subsets, polarisation and ontogeny, inflammasome regulation, and the role of non-canonical inflammasomes, NK cells, NETosis, specialised pro-resolving mediators, neurotransmitters, and rarer T cell subsets. ScRNA-Seq with or without single-cell proteomics can be used to investigate these areas in addition to other ‘omics’ modalities. Adults, children, HIV-uninfected and PLWH must be proportionally represented given the acknowledged differences in their immune phenotypes. Longitudinal studies allow an insight into dynamic changes in the immune response, especially relevant in the context of paradoxical reactions and TBM-IRIS. The path forward must also maintain a translational focus ensuring that any advances made lead to rapid improvement in outcomes for patients diagnosed with this devastating disease.

## Author contributions

JB: Writing – original draft. AD: Writing – review & editing. RW: Writing – review & editing.
